# Speckle Tracking Based Strain Analysis Is Sensitive for Early Detection of Pathological Cardiac Hypertrophy

**DOI:** 10.1371/journal.pone.0149155

**Published:** 2016-02-12

**Authors:** Xiangbo An, Jingjing Wang, Hao Li, Zhizhen Lu, Yan Bai, Han Xiao, Youyi Zhang, Yao Song

**Affiliations:** Institute of Vascular Medicine, Peking University Third Hospital, Key Laboratory of Cardiovascular Molecular Biology and Regulatory Peptide, Ministry of Health, Beijing Key Laboratory of Cardiovascular Receptors Research, Beijing, 100191, China; Emory University, UNITED STATES

## Abstract

Cardiac hypertrophy is a key pathological process of many cardiac diseases. However, early detection of cardiac hypertrophy is difficult by the currently used non-invasive method and new approaches are in urgent need for efficient diagnosis of cardiac malfunction. Here we report that speckle tracking-based strain analysis is more sensitive than conventional echocardiography for early detection of pathological cardiac hypertrophy in the isoproterenol (ISO) mouse model. Pathological hypertrophy was induced by a single subcutaneous injection of ISO. Physiological cardiac hypertrophy was established by daily treadmill exercise for six weeks. Strain analysis, including radial strain (RS), radial strain rate (RSR) and longitudinal strain (LS), showed marked decrease as early as 3 days after ISO injection. Moreover, unlike the regional changes in cardiac infarction, strain analysis revealed global cardiac dysfunction that affects the entire heart in ISO-induced hypertrophy. In contrast, conventional echocardiography, only detected altered E/E’, an index reflecting cardiac diastolic function, at 7 days after ISO injection. No change was detected on fractional shortening (FS), E/A and E’/A’ at 3 days or 7 days after ISO injection. Interestingly, strain analysis revealed cardiac dysfunction only in ISO-induced pathological hypertrophy but not the physiological hypertrophy induced by exercise. Taken together, our study indicates that strain analysis offers a more sensitive approach for early detection of cardiac dysfunction than conventional echocardiography. Moreover, multiple strain readouts distinguish pathological cardiac hypertrophy from physiological hypertrophy.

## Introduction

Cardiac hypertrophy is a generic response of the myocardium to various physiological and pathophysiological stimuli, characterized by increased cardiac mass relative to body weight. Hypertrophy is broadly divided into two categories: adaptive and maladaptive. Adaptive hypertrophy involves physiological cardiac hypertrophy induced by physiological stimuli, such as exercise and pregnancy, and compensated hypertrophy in response to hemodynamic stress, neurohumoral stimuli and other pathological insults. [1, 2] Physiological hypertrophy is characterized by increased cardiac size with normal and/or enhanced cardiac function. In particular, exercise-induced physiological hypertrophy provides substantial cardioprotection against ischemia-reperfusion injury and pressure overload insult. [3, 4] Upon pathological stimuli, compensated hypertrophy is initially adaptive and beneficial, in that the increase in ventricular wall thickness normalizes increased wall tension to maintain normal cardiac function. However, if the pathological stimuli sustain, such as unresolved hemodynamic stress or neurohumoral over-stimulation, compensated hypertrophy may progress to maladaptive hypertrophy and heart failure. [5] Therefore, it is critical to prevent or reverse the pathological hypertrophic phenotype at an early stage to circumvent the subsequent development of heart failure.

Unfortunately, due to the lack of specific clinical features, detection and diagnosis of pathological cardiac hypertrophy at early stages are difficult, which often lead to the loss of optimal opportunity for treatment. Conventional echocardiography is the most commonly used approach for diagnosing heart diseases, due to its convenience, cost-effectiveness, non-invasiveness, and availability for bedside examination. [6, 7] In particular, echocardiography is powerful for identification of geometrical changes and explicit dysfunction arising from heart remodeling. However, owing to well compensated cardiac function at the early stages of pathological hypertrophy, conventional echocardiography often fails in detecting abnormal cardiac performance and distinguishing pathological hypertrophy from physiological hypertrophy. Thus, new diagnostic methods that may overcome the aforementioned limitations are in urgent need. Speckle tracking based strain analysis is a recently-developed tool derived from 2D cine loop imaging of ultrasound. Given the high levels of reproducibility, quantitative capability and user friendly features, strain and strain rate have become cutting-edge tools for detecting cardiac performance. An increasing volume of clinical data suggest that strain and strain rate are advantageous in early detection and prognosis of myocardial infarction [8] and in differentiating transmural from non-transmural myocardial infarction. [9] These discriminative parameters are also more advantageous for assessing the recovery of regional function after ST-segment elevation myocardial infarction in patients undergoing percutaneous coronary intervention. [10] These findings have provided strong evidence that strain and strain rate are useful and sensitive parameters in assessing cardiac performance.

Small animal models for cardiac hypertrophy are important tools for understanding pathological mechanisms and developing therapeutic strategies for the treatment and prevention of heart diseases. However, to date, the application of strain imaging in small animal models is still limited because the imaging acquisition designed for humans is not suitable for mice. In this study, we used VevoStrain software designed for the Vevo 2100 system, which is able to achieve higher resolution at up to 30 μm, in contrast to 200–300 μm for human, to measure myocardial performance of two types of mouse models, pathological hypertrophy caused by over-activation of β-AR and physiological hypertrophy induced by running exercise, to verify if speckle tracking based strain analysis is more sensitive compared to conventional echocardiography for identifying cardiac dysfunction induced by over-activation of β-AR at early stages and if this tool could differentiate pathological cardiac hypertrophy from physiological hypertrophy.

## Materials and Methods

The investigations conformed to the US National Institutes of Health Guide for the Care and Use of Laboratory Animals (NIH Publication No. 85–23, revised 1996). All the experiments were approved by Peking University Institutional Committee for Animal Care and Use. Mice were kept under standard pathogen-free conditions with a standard diet and regular 12: 12 light-dark cycle. Male C57BL/6 mice (10 weeks old) were provided by the Animal Department of Peking University Health Science Center (Beijing).

### Mouse models

Mice were subjected to normal saline (control), acute over-activation of β-AR and running exercise, respectively. The models with acute over-activation of β-AR were induced by a single injection (5mg/kg body weight, subcutaneously) of isoproterenol (ISO, Sigma-Aldrich, St. Louis, MO, USA), a β-adrenergic receptor agonist. The model of physiological cardiac hypertrophy was established by long-term running exercise. In brief, the mice were trained by treadmill and their maximal oxygen consumption (VO_2max_) was detected by respiratory metabolism system (O_2_/CO_2_ Analyzer, Panlab-HARVARD APPARATUS, LE405, Spain). The velocity of 80% VO_2max_, 15cm/s, was used as a standard parameter for training the mice. The mice were exercised for one and a half hour every day and one day free every week. Cardiac geometry and function were detected by conventional echocardiography and VevoStrain software at 3 days and 7 days after ISO injection, and also six weeks after running exercise. Then the mice were sacrificed under anesthesia with 2% isoflurane, and the ratios of heart weight (HW) to tibia length were determined and calculated.

### Conventional echocardiographic measurements

Cardiac geometry, systolic and diastolic function were evaluated by echocardiography using a VISUALSONICS high-resolution Vevo 2100 system (VISUALSONICS Inc., Toronto, Canada) equipped with a 30-MHz transducer. Briefly, mice were placed in supine position on a movable, heated platform maintained at 37°C, and anesthetized with 1.0%-1.5% isoflurane (Baxter Healthcare Corp, New Providence, RI, USA) to keep the heart rate stabilized at 400 to 500 beats per minute. Conventional echocardiographic parameters, such as wall thickness and chamber dimensions, were obtained from M-mode images at the mid-papillary level in the parasternal short axis view, and also from B-mode images acquired in the parasternal long- and short-axis views, then EF (%) and FS (%) were calculated. Transmitral flow measurements of ventricular filling velocity were obtained from apical four-chamber view using pulsed Doppler, so the early diastolic (E), the late diastolic (A), and the ratio E/A were obtained to assess diastolic function. Besides, the early-diastolic peak velocity (E’), the late-diastolic peak velocity (A’) of mitral valve ring and E’/A’ were also obtained from this view. Thus E/E’ was calculated, which is also a parameter reflecting diastolic function.

### Echocardiographic speckle tracking based strain analysis of myocardial deformation

**Fig 1:**
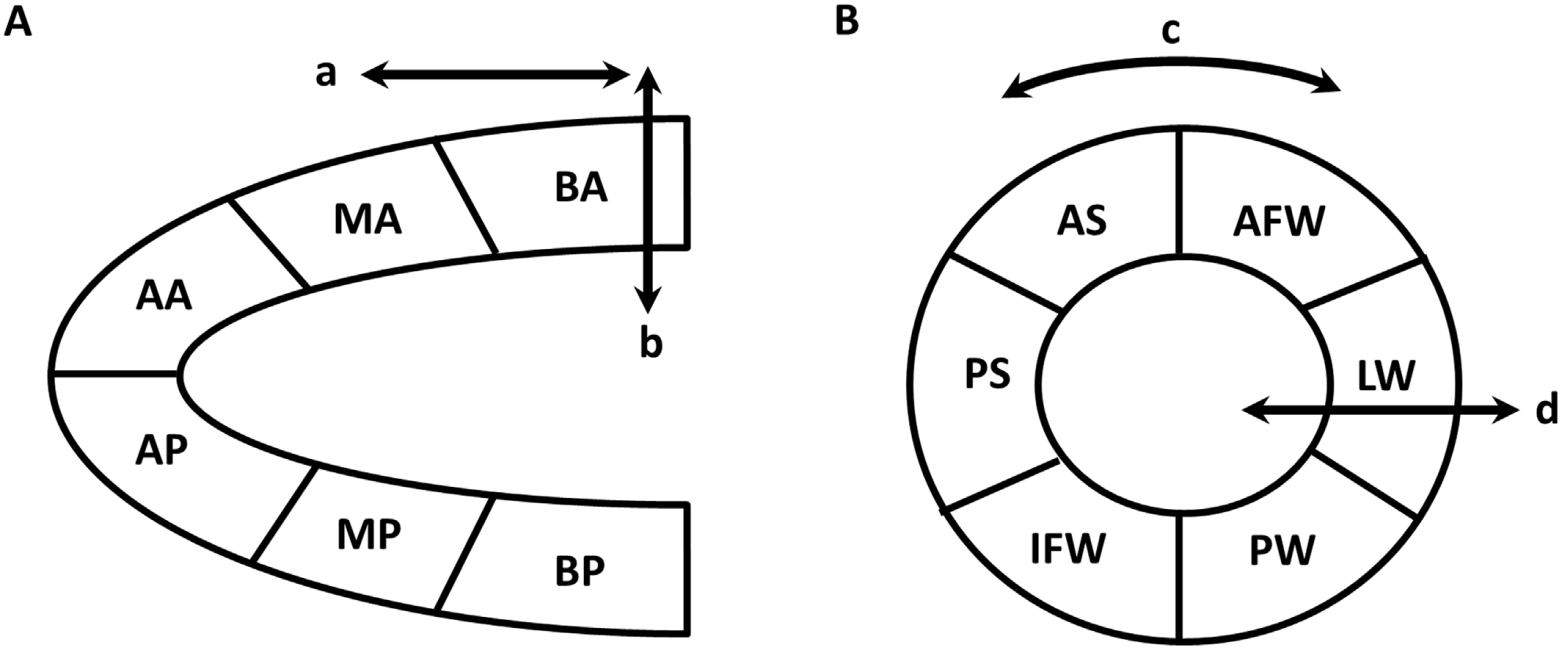
Schematic diagram of speckle tracking-based strain analysis. Strain analysis is based on speckle tracking, which uses acoustic back scatter on 2D grayscale ultrasound images as tissue marker. Cardiac function is defined by myocardial deformation along the longitudinal, radial, and circumferential axes, according to myocardial fiber orientation. (A) Parasternal long-axis view provides longitudinal (arrow a) and radial (arrow b) strain and strain rate. (B) Circumferential (arrow c) and radial (arrow d) strain and strain rate are obtained from parasternal short-axis view. Both long- and short-axis views are divided automatically into six segments for speckle tracking throughout the cardiac cycle. BA indicates basal anterior; MA, mid-anterior; AA, apical anterior; BP, basal posterior; MP, mid-posterior; AP, apical posterior; AFW, anterior free wall; LW, lateral wall; PW, posterior wall; IFW, inferior free wall; PS, posterior septum; AS, anterior septum.

Strain analysis is based on combined speckle tracking algorithms applied on high-frequency ultrasound images. [11] By definition, strain indicates how much the myocardial tissue has deformed, i.e. *Strain (S) = ΔL/L*_*0*_ *= (L*_*1*_*-L*_*0*_*)/L*_*0*_, and strain rate (SR) reflects how fast the myocardial tissue is deforming, i.e. *SR = S/Δt = ΔL/L*_*0*_*/Δt*. [12] Therefore, strain and SR can reflect the regional and global systolic and diastolic function. Strain and SR were quantified in the longitudinal, radial, and circumferential axes by speckle tracking of 2D grayscale echocardiographic images acquired from the parasternal long- and short-axis views. VevoStrain analyses were conducted by the same trained investigator on all animals using VisualSonics 2100. In brief, B-mode cine loops were selected from 2D echocardiographic images, and then three to four consecutive cardiac cycles were selected to analyze the strain and SR. Tracing of the endocardial borders was performed. Then the strain and SR were analyzed by Vevo2100 workstation so that regional and global measures were acquired, respectively. [8] Long axis or short axis view of the left ventricle (LV) myocardium was automatically divided into six segments for regional speckle tracking. The peak of regional strain and SR are obtained from the six standard segments. At the same time, the global peak of strain and SR were also acquired (Fig 1).

### Histological analysis

Following anesthesia with intraperitoneal sodium pentobarbital (100 mg/kg), the mice were sacrificed after pedal pinch reflex were completely inhibited. Hearts were arrested in diastole, fixed with 4% paraformaldehyde and paraffin embedded. Then the hearts were sectioned at 5 μm, and stained with hematoxylin and eosin (H&E), and picrosirius red. The morphometric features of hearts were evaluated by Leica Q550 IW imaging workstation (Leica Microsystems Imaging Solutions, Cambridge, UK). The H&E images of left ventricle sections were taken under x400 magnification. Cross-sectional areas of cardiomyocytes were measured by Leica Q550 IW Imaging Workstation. Fifty cells of each section from four random views were calculated. The picrosirius red staining images of heart were taken under x1.25 magnification and analyzed by Image-Pro Plus 6.0. Therefore the percentage of cardiac fibrosis area was calculated by the ratios of collagen area to myocardial area.

### Statistical analysis

All data were expressed as mean ± SD. Inter-group comparisons were conducted using one-way ANOVA with Tukey-post-test or two-way ANOVA with Bonferroni-post-test (Prism 5, GraphPad Software Inc., La Jolla, CA, USA). P<0.05 was considered statistically significant.

## Results

### Speckle tracking is more sensitive in detecting isoproterenol-induced acute cardiac dysfunction at earlier stages than conventional echocardiography

**Fig 2:**
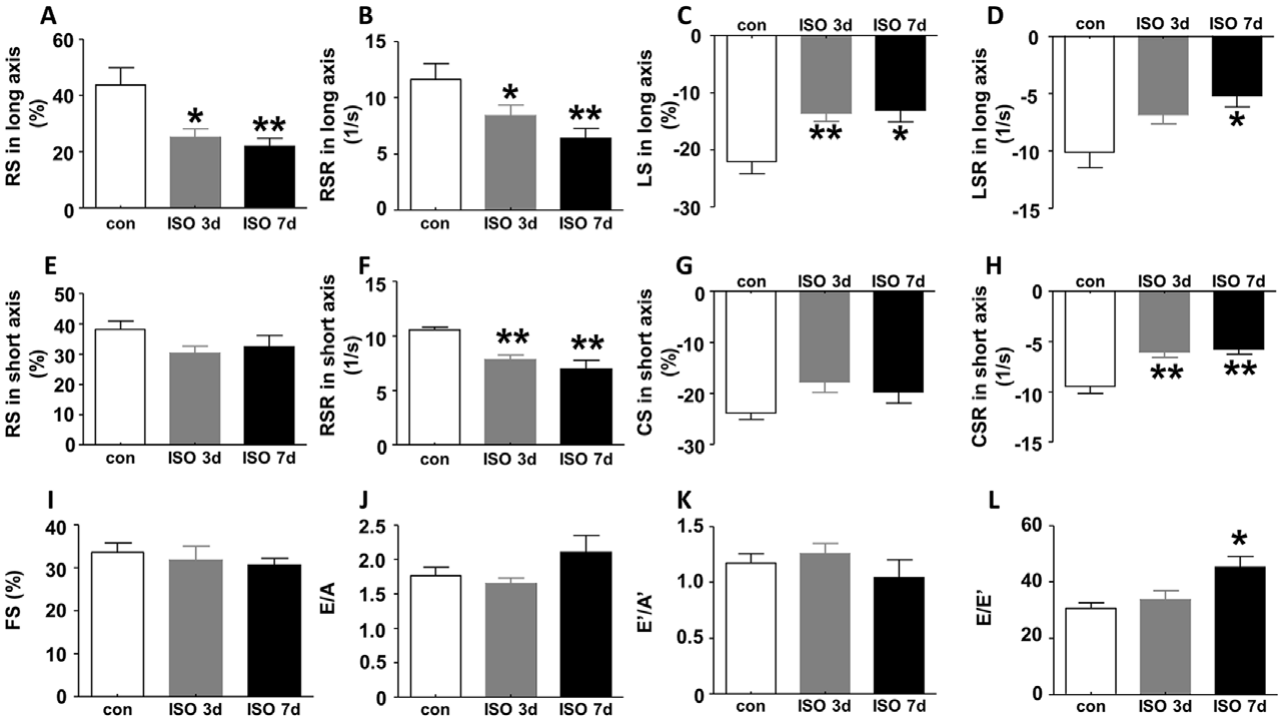
Strain analysis and conventional echocardiographic assessment of LV function following ISO insult. In parasternal long axis, (A) global radial strain (RS), (B) radial strain rate (RSR) and (C) longitudinal strain (LS) decreased both at 3 days and 7 days after ISO injection, and (D) longitudinal strain rate (LSR) decreased at 7 days after ISO injection. In short axis, (E) global RS and (G) circumferential strain (CS) only showed a trend of decrease at 3 days and 7 days after ISO injection, but (F) RSR and (H) circumferential strain rate (CSR) both decreased at the two time point following ISO insult. However, there was no difference in (I) FS, (J) E/A and (K) E’/A’ either at 3 days or at 7 days after ISO injection, and (L) E/E’ only increased at 7 days after ISO treatment compared with control. Data are shown as mean ± SD, and statistically analyzed using one-way ANOVA among mice subjected to ISO treatment and control. n = 6 per group, *P<0.05, **P<0.01, compared with control. FS, fractional shortening; con, control; ISO, isoproterenol.

Strain is a dimensionless parameter that indicates how much the myocardial tissue has deformed and strain rate (SR) reflects how fast the myocardial tissue is deforming. [12] Therefore, strain and SR can reflect the regional and global systolic and diastolic function. In our speckle tracking-based strain analysis, cardiac function is defined by myocardial deformation along the longitudinal, radial, and circumferential axes as illustrated in Fig 1. Speckle tracking uses acoustic back scatter on 2D grayscale ultrasound images as tissue marker. 2D echocardiographic cine loops and M-mode images were acquired at the similar heart rate. As shown in Fig 2, multiple parameters, including radial strain (Fig 2A), radial strain rate (Fig 2B) and longitudinal strain (Fig 2C), were significantly reduced 3 and 7 days after ISO treatment. In addition, longitudinal strain rate (LSR), an assessment by speckle tracking based strain analysis in long axis, was also significantly reduced at 7 days after ISO injection (Fig 2D). In parasternal short axis, RS and circumferential strain (CS) showed a trend of decrease upon ISO insult (Fig 2E and 2G), whereas both RSR and circumferential strain rate (CSR) dramatically decreased at 3 days and 7 days after ISO injection compared with control (Fig 2F and 2H). In contrast, conventional echocardiography did not detect any abnormalities in FS, a measurement for LV systolic function after ISO injection (Fig 2I). Regarding diastolic function, the common parameter, E/A, did not show obvious difference between ISO-treated mice and control (Fig 2J) and neither did E’/A’ (Fig 2K). The only parameter of conventional echocardiography that showed abnormality is E/E’, an index that measures diastolic function, which was increased at 7 days after ISO treatment compared with control but did not change at 3 days after ISO injection (Fig 2L).

### Temporary ISO insult results in a global rather than regional cardiac dysfunction detected by strain analysis

**Fig 3:**
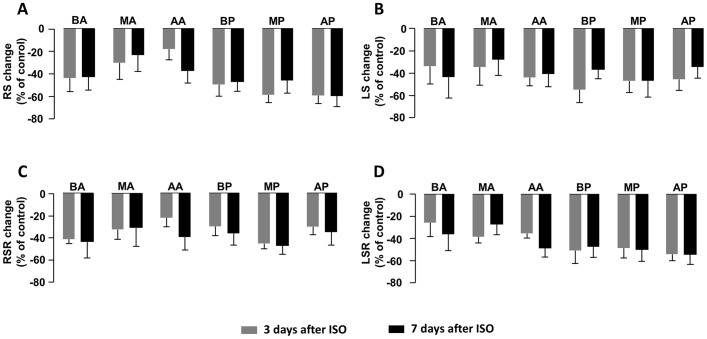
Comparison of changes in regional strain and strain rate after ISO insult. (A) RS, (B) LS, (C) RSR and (D) LSR in long axis all decreased in each segment of LV at 3 days and 7 days after ISO injection compared with control, however, the change rates of the above parameters did not show any significant difference among the six segments. Data are shown as percentage of changes versus control following ISO injection. Two-way ANOVA is used to compare statistical difference in change rates among the six segments. ISO, isoproterenol; BA, basal anterior; MA, mid-anterior; AA, apical anterior; BP, basal posterior; MP, mid-posterior and AP, apical posterior. n = 6 per group.

Left ventricle (LV) was divided into six segments in parasternal long axis: basal anterior (BA), mid-anterior (MA), apical anterior (AA), basal posterior (BP), mid-posterior (MP) and apical posterior (AP). To investigate if ISO-induced heart injury was global or regional, we measured strain and SR of each segment and compared the change rate relative to control among six segments. As shown in Fig 3, the strain and SR of all segments decreased to similar degrees as compared with the corresponding controls, including RS (Fig 3A), LS (Fig 3B), RSR (Fig 3C) and LSR (Fig 3D). Therefore, unlike the previously reported regional abnormalities in myocardial infarction detected by strain analysis, [13] ISO induced a global decrease in strain and SR, suggesting an overall cardiac dysfunction.

### Strain analysis, but not conventional echocardiography, can be used to differentiate ISO-induced cardiac hypertrophy from exercise-induced physiological cardiac hypertrophy

**Fig 4:**
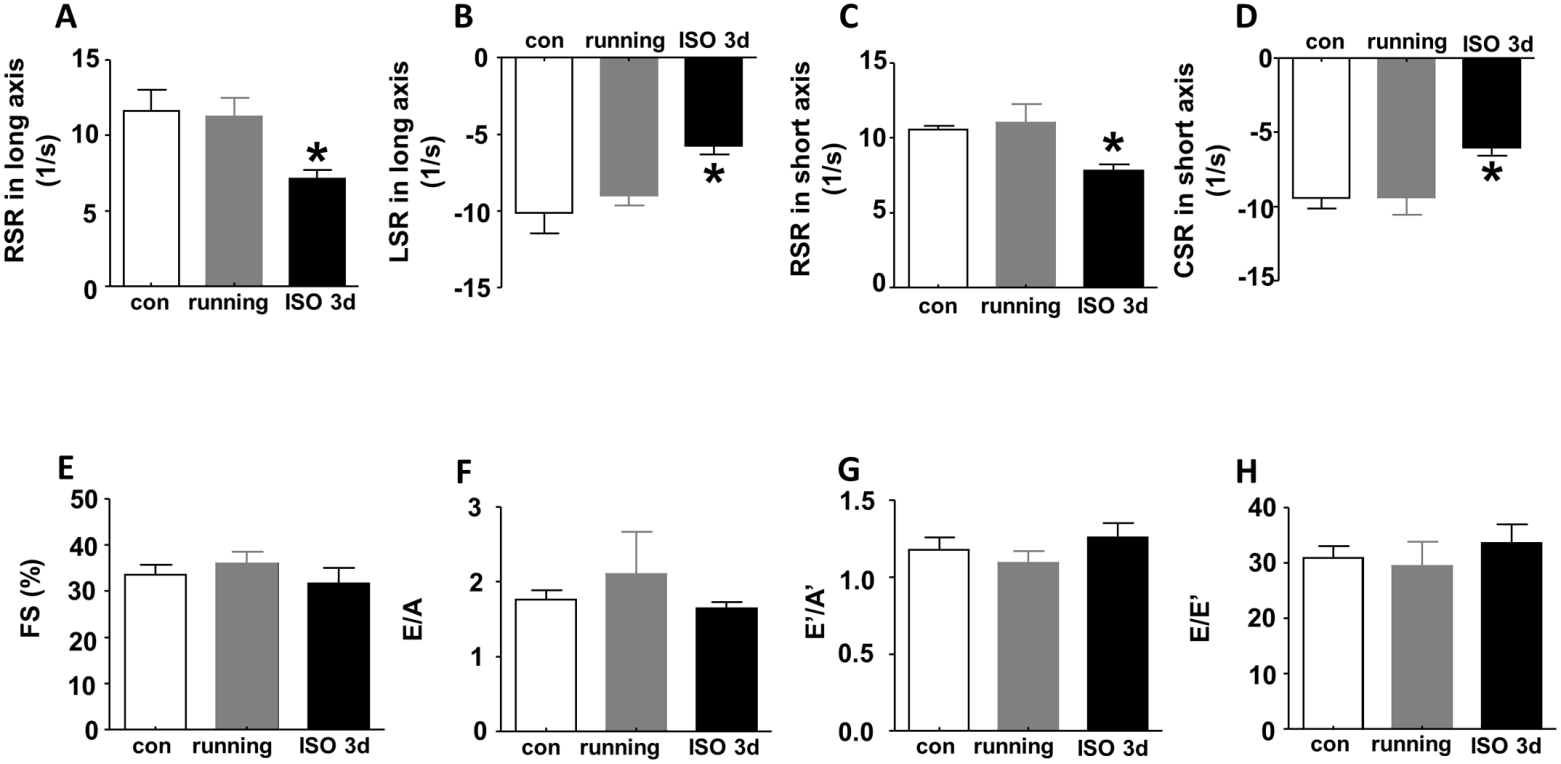
Superiority of strain analysis to conventional echocardiography in differentiating ISO-induced cardiac hypertrophy from running exercise-induced physiological cardiac hypertrophy. (A) Global RSR, (B) LSR in long axis and (C) RSR, (D) CSR in short axis decreased in ISO-induced cardiac hypertrophy but not in running-induce cardiac hypertrophy compared to control. However, (E) FS%, (F) E/A, (G) E’/A’ and (H) E/E’ showed no difference among ISO insult, running and control. Data are shown as mean ± SD, and one-way ANOVA is used for comparing statistical difference among control, running and ISO insult. n = 6 per group, *P<0.05, compared with control. FS, fractional shortening; con, control; ISO, isoproterenol.

We next questioned whether strain analysis and/or the conventional echocardiography could distinguish the pathological changes caused by ISO-induced cardiac hypertrophy from physiological hypertrophy. To achieve this goal, we established a mouse model of physiological cardiac hypertrophy through daily treadmill running exercise for 6 weeks. We performed speckle tracking and conventional echocardiography on two groups of mice that were subjected to ISO-insult or running exercise, respectively. We found that global RSR and LSR of LV obtained from parasternal long axis were significantly decreased in ISO-induced cardiac hypertrophy compared with control. In contrast, no alterations were detected in mice that developed hypertrophy following running exercise (Fig 4A and 4B). In addition, RSR and CSR in short axis were also reduced upon ISO insult compared with control, but not altered after running exercise (Fig 4C and 4D). However, conventional echocardiographic parameters did not show any statistical differences among control, running and ISO insult. As shown in Fig 4E–4H, there was no difference in FS%, E/A, E’/A’, and E/E’ between running, ISO treatment when compared with control. These results demonstrate that strain analysis, but not the conventional echocardiography, can distinguish running exercise-induced physiological cardiac hypertrophy from pathological cardiac hypertrophy caused by ISO-induced β-AR insult at early stages.

### ISO-induced cardiac hypertrophy display pathological manifestations that are distinct from running exercise-induced cardiac hypertrophy

**Fig 5:**
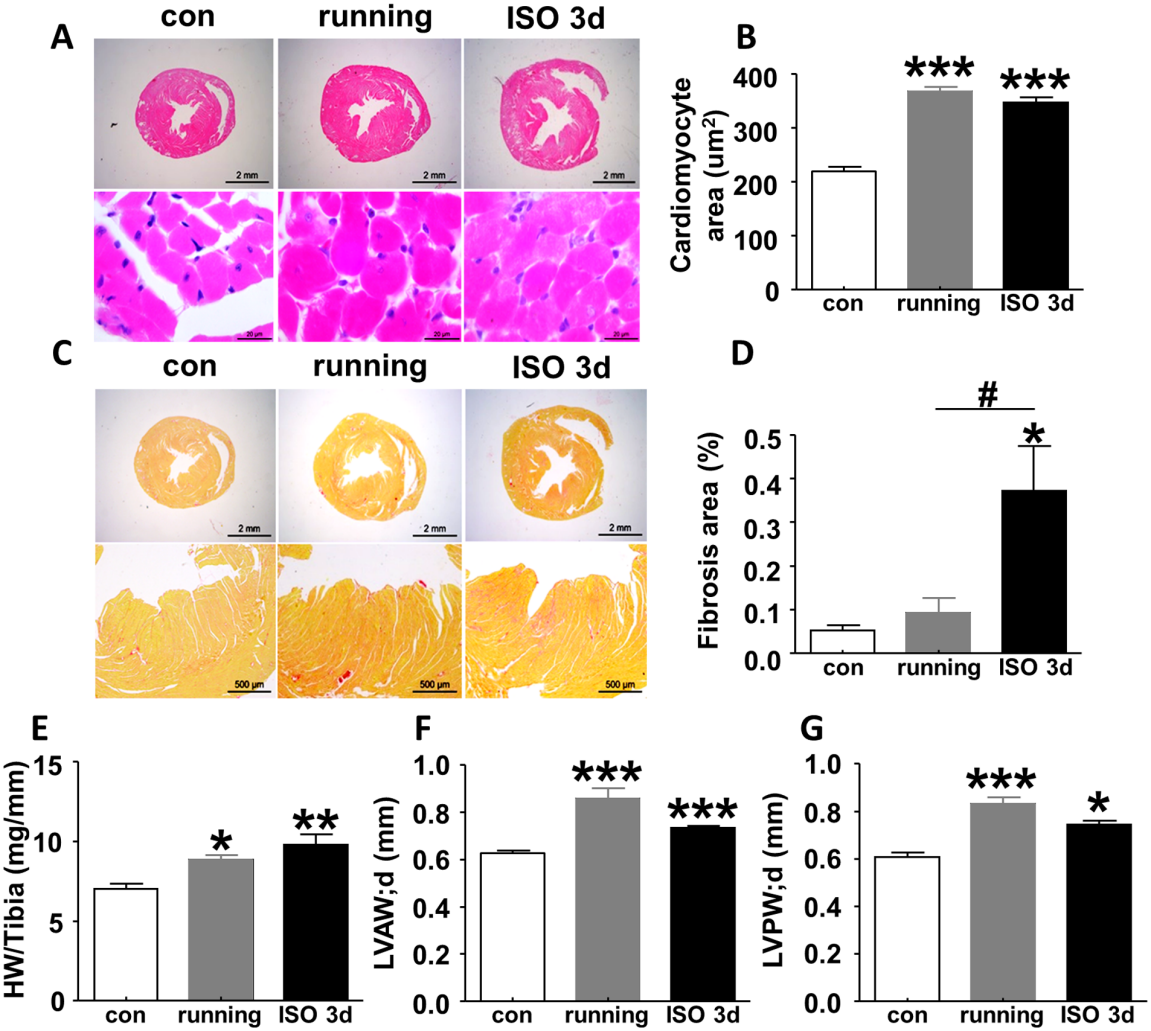
Characteristics of heart pathology in mice following 6-week running exercise and ISO insult. (A, B) Heart cross-sectional HE staining showed cardiomyocytes enlarged in a uniform manner both in exercised mice and in ISO-treated mice as compared with control. (C, D) Picrosirius red staining indicated collagen deposit increased at 3 days after ISO injection but did not change following running exercise. (E) Ratio of heart weight to tibia length (HW/TL), (F) LVAW;d and (G) LVPW;d remarkably increased in mice subjected to running and ISO insult vs. control mice, suggesting both 6-week running exercise and single-dose ISO injection resulted in cardiac hypertrophy. Data are shown as mean ± SD, and one-way ANOVA is used to analyze the statistical difference among control, running and ISO insult. n = 6 per group, *P<0.05, **P<0.01, ***P<0.001, compared with control; ^#^P<0.05, compared with running. LVAW;d, left ventricular anterior wall thickness in diastolic; LVPW;d, left ventricular posterior wall thickness in diastolic; con, control; ISO, isoproterenol.

To delineate the pathological basis for the abnormal strain parameters selectively found upon ISO-induced β-adrenergic insult, we compared the degree of cardiac hypertrophy and collagen deposit in mice subjected to ISO-treatment or running exercise, respectively. As shown in Fig 5A and 5B, HE staining indicated comparable cardiomyocytes enlargement in a uniform manner both in mice subjected to running exercise and ISO injection compared with control. In addition, both running exercise and ISO treatment led to remarkable increase in the ratio of heart weight to tibia length (HW/TL) relative to control, which is the gold standard for cardiac hypertrophy (Fig 5C). Moreover, echocardiographic measurements indicated comparable changes of the left ventricular anterior wall thickness in diastolic (LVAW;d, Fig 5D) and left ventricular posterior wall thickness in diastolic (LVPW;d, Fig 5E) caused by running exercise and ISO insult compared with control. These data demonstrate similar levels of hypertrophy in our physiological and pathological paradigms. However, picrosirius red staining showed disperse but obvious collagen deposit arose only in myocardium 3 days after ISO injection. In contrast, no obvious collagen formation was detected in the myocardium of control mice or mice subjected to running exercise (Fig 5F and 5G). This result suggests that collagen formation in myocardium, which is indicative of fibrosis and pathological remodeling that increases the stiffness of the heart, is an underlying pathological alterations that for the reduced strain parameters.

## Discussion

In the present study, we demonstrated that speckle tracking-based strain analysis was more sensitive than conventional echocardiography for early detection of pathological cardiac hypertrophy induced by ISO, and could distinguish the pathological cardiac hypertrophy from physiological cardiac hypertrophy induced by running exercise.

Rapid over-activation of β-adrenergic receptor usually triggers pathological reactions and leads to heart damage, which may develop to cardiac remodeling. In our study, histological staining showed that cardiomyocyte size and collagen deposit increased in myocardium at 3 days following ISO insult, indicating that rapid over-activation of β-AR was sufficient to cause substantial cardiac remodeling. To date, diverse approaches have been applied to diagnose cardiac remodeling, including magnetic resonance imaging (MRI), positron emission tomography/computed tomography (PET/CT) and echocardiography etc. MRI sequences help to detect LV function, regional perfusion, angiogenesis, myocardial viability and orientations of myocytes. Diffusion MRI helps to provide information of microscopic tissue structure. [14] Lapa C et al. found that somatostatin receptor (SSTR) based PET/CT could detect cardiac inflammation after acute myocardial infarction or acute peri-/myocarditis. This approach would become a new potential biomarker in predicting cardiac remodeling. [15] Recently some new approaches have been developed to discover cardiac dysfunction at an early time point. Diffusion tensor imaging (DTI) provides a valuable non-destructive tool for characterizing structure remodeling in diseased myocardium. [16] Molecular imaging, such as imaging of avB3 (alpha v beta 3) integrin has been used to noninvasively visualize angiogenesis in infarcted hearts. [17] Quantitative PET imaging has been applied to detect early metabolic remodeling in a mouse model of left ventricular hypertrophy induced by transverse aortic construction (TAC). [18] Yet application of these approaches is quite limited since they are costly, inconvenient, not real-time, and over-dependent on contrast agents. In contrast, speckle tracking based strain analysis offers advantages to overcome the above limitation and becomes more and more commonly used to evaluate cardiac function clinically. [8, 9, 19] It has been verified to be of great value in the early detection of trastuzumab and anthracycline mediated cardiomyopathy. [20]

In the present study, we used strain analysis to evaluate cardiac function following rapid β-AR over-activation through VevoStrain software newly developed for Vevo 2100 ultrasound system. It indicated that RS, RSR and LS in long axis as well as RSR and CSR in short axis decreased as early as 3 days after ISO injection. However, conventional echocardiography was unable to detect cardiac dysfunction at 3 days after ISO injection, only E/E’ appeared abnormal at 7 days after ISO injection. E/E’ is derived from Tissue Doppler and has been regarded as a good index for assessing cardiac diastolic dysfunction, [21] which seems to be more sensitive than the other conventional echocardiographic parameters in our study. Nevertheless it still can be seen that speckle tracking is more sensitive for diagnosing cardiac dysfunction than conventional echocardiography. We then demonstrated that this type of cardiac dysfunction caused by a transient and relatively lower dosage of ISO treatment was global rather than regional because strain and strain rate from each segment of LV decreased homogeneously. Given that strain analysis is based on the measurement of six individual segments, it is advantageous over other parameters in discriminating global and regional dysfunction. Thibault H et al. [13] reported that SR mainly decreased in mid-anterior, apical posterior and apical anterior wall, and it could differentiate transmural from nontransmural and noninfarcted myocardium not only in acute stage but also in chronic phase following myocardial infarction caused by left coronary artery ligation. Nevertheless Bauer M and colleagues [8] found myocardial performance, as represented by LS, decreased both in the infarct segments and remote regions though apical anterior and apical inferior wall were more severe. This discrepancy may be involved in the different severity of myocardial damage.

Furthermore, our findings also indicated that strain and SR well reflected the pathology of the heart, since the global decreases in strain and SR were in consistence with the features of heart cross-sectional histological staining in pathological cardiac hypertrophy, which showed uniformly enlarged cardiomyocytes and disperse collagen deposit without local necrosis. In contrast to pathological hypertrophy, exercise-induced physiological hypertrophy only represents an increase in cardiac mass and cardiomyocyte dimension, but does not lead to fibrosis, which is regarded as the most marked difference from pathological hypertrophy in pathology. [22] Our study demonstrated that 6-week running exercise caused cardiomyocyte enlargement but with no sign of fibrosis, which was indicative of a typical physiological cardiac hypertrophy. Interestingly, strain and strain rate did not change in physiological hypertrophic heart as compared with control, which is distinct from pathological cardiac hypertrophy. As a matter of fact, pathological cardiac hypertrophy usually indicates normal cardiac function at compensated stage as determined by traditional approaches, which makes it difficult to tell whether a case with cardiac hypertrophy is pathological or physiological. In this respect, our findings have provided a line of evidence that strain analysis could be an ideal measure for distinguishing the pathological cardiac hypertrophy from physiological cardiac hypertrophy at an early stage.

## Conclusions

Taken together, speckle tracking based strain analysis is more sensitive for early diagnosis of pathological cardiac hypertrophy induced by temporary ISO insult compared with conventional echocardiography, and it seems to be a promising tool for distinguishing pathological cardiac hypertrophy from physiological cardiac hypertrophy at early stages.
